# Author Correction: Large-scale resculpting of cortical circuits in children after surgical resection

**DOI:** 10.1038/s41598-021-88186-8

**Published:** 2021-04-13

**Authors:** Anne Margarette S. Maallo, Michael C. Granovetter, Erez Freud, Sabine Kastner, Mark A. Pinsk, Daniel Glen, Christina Patterson, Marlene Behrmann

**Affiliations:** 1 Department of Psychology and Neuroscience Institute, Carnegie Mellon University, Pittsburgh, USA; 2 School of Medicine, University of Pittsburgh, Pittsburgh, USA; 3 Department of Psychology, The Centre for Vision Research, York University, Toronto, Canada; 4 Princeton Neuroscience Institute, Princeton University, Princeton, USA; 5 Department of Psychology, Princeton University, Princeton, USA; 6 Scientific and Statistical Computing Core, National Institute of Mental Health, Bethesda, USA; 7 Department of Pediatrics, University of Pittsburgh, Pittsburgh, USA

Correction to: *Scientific Reports* 10.1038/s41598-020-78394-z, published online 09 December 2020

Daniel Glen was omitted from the author list in the original version of this Article. This has been corrected in the PDF and HTML versions of the Article, and in the accompanying Supplementary Information file.

The Author Contributions section now reads:

“A.M.S.M.: conception and design; acquisition, analysis, and interpretation of data; created new code to use in work; wrote and revised manuscript; M.C.G.: acquisition, analysis, and interpretation of data; created new code to use in work; revised manuscript; E.F.: analysis, and interpretation of data; created new code to use in work; revised manuscript; S.K.: analysis, and interpretation of data; revised manuscript; M.A.P.: analysis, and interpretation of data; D.G.: created new code to use in work; C.P.: contributed unpublished tools; recruited and managed patients; revised manuscript M.B.: conception and design; interpretation of data; wrote and revised manuscript.”

The Acknowledgements section now reads:

“The authors would like to thank the participants and their families for taking part in this study. We also would like to thank Ms Jennifer Monahan of Children’s Hospital for assistance in patient recruitment, and Mr Scott Kurdilla and Mr Mark Vignone for assistance in scanning the participants. We also thank Dr Avital Hahamy and Dr David Plaut for useful suggestions and conversations. This research was supported by grants from the National Eye Institute (NIH) RO1 EY0207018 to MP and CP, from the National Institute of General Medical Sciences (NIH) T32 GM081760 to MCG, and from the Intramural Research Programs of the National Institute of Mental Health and National Institute of Neurological Disorders and Stroke (NIH) ZICMH002888 and by the Brain Initiative (NIH) 1R24MH117467-01 to DG. The content is solely the responsibility of the authors and does not necessarily represent the official views of the National Eye Institute, National Institute of General Medical Sciences, National Institute of Mental Health, National Institute of Neurological Disorders and Stroke, or the National Institutes of Health.”

